# Chronic Melioidosis Presenting with Pneumonia, Empyema, and Splenic Abscess

**DOI:** 10.4269/ajtmh.26-0143

**Published:** 2026-08-11

**Authors:** Yamei Zheng, Xiaozhou Yao, Min Wang, Tian Xie

**Affiliations:** ^1^ Department of Pulmonary and Critical Care Medicine, Hainan General Hospital (Hainan Affiliated Hospital of Hainan Medical University), Hainan, China;; ^2^ Department of Pharmacy, Hainan General Hospital (Hainan Affiliated Hospital of Hainan Medical University), Hainan, China

## Abstract

Melioidosis is an emerging tropical infectious disease with diverse clinical manifestations and a high fatality rate. We report a rare case of chronic melioidosis manifesting as pulmonary infection, empyema, and splenic abscess. The patient presented with recurrent chest pain, cough, and abdominal discomfort for 11 months, initially misdiagnosed as common bacterial infection. Computed tomography imaging revealed pulmonary infection, left pleural effusion, and multiple abnormal low-density lesions in and around the spleen. Pleural effusion culture identified *Burkholderia pseudomallei.* Management included thoracic puncture and catheter drainage, an intensive phase of intravenous ceftazidime, followed by an eradication phase with oral trimethoprim/sulfamethoxazole. The patient showed clinical and radiological improvements. Clinicians should consider chronic melioidosis in patients with pneumonia and parapneumonic pleural effusion accompanied by splenic abscesses, particularly in patients with diabetes mellitus from endemic areas. Early recognition, definitive diagnosis, and tailored antimicrobial therapy are essential to prevent the progression of severe pneumonia and sepsis.

## INTRODUCTION

Melioidosis is an under-recognized fatal and zoonotic disease caused by *Burkholderia pseudomallei*.^1^ Geographic and climatic factors primarily determine the survival and reproduction of *B. pseudomallei*. Hot and humid conditions in tropical and subtropical regions, such as Southeast Asia and Australia, provide a favorable environment for the growth of *B. pseudomallei*.^2^ In China, the highest incidence is mainly concentrated in southern cities, such as Guangdong, Hainan, and Guangxi. Melioidosis is a severe emerging infectious disease with a high mortality rate of up to 70%.^3^ The clinical presentation of melioidosis can be acute (approximately 85%), subacute, or chronic (usually less than 12%).^4^ Acute disease is characterized by the presence of symptoms for less than 2 months and usually manifests as pneumonia with severe sepsis. Chronic infection is defined as the presence of symptoms for over 2 months, similar to tuberculosis.^5^ Studies have indicated that the principal clinical manifestations of chronic melioidosis are abscesses at multiple sites, which may present with fever of unknown origin. Furthermore, persistent infectious skin ulcers have rarely been reported as clinical manifestations of chronic melioidosis.^6^ Herein, we present a case of chronic melioidosis manifesting as recurrent pulmonary infections, pleural effusion, and splenic abscesses. This clinical presentation is uncommon and follows a prolonged and insidious course. Melioidosis was confirmed based on pleural fluid culture, and the patient reported in this study was cured following standardized anti-infective therapy. The details are described in the subsequent sections.

## CASE PRESENTATION

**Figure 1.**
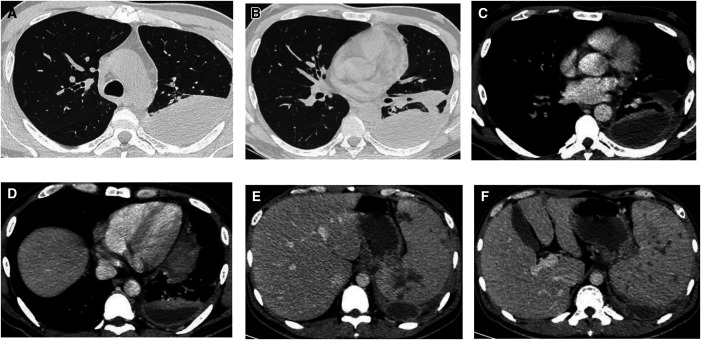
Initial computed tomography of the thoracic and abdominal regions. (**A–D**) Infectious lesions in the left lung, primarily in the lower lobe, with partial consolidation and left-sided encapsulated pleural effusion. (**E**, **F**) Weakly enhanced shadows in the spleen and a low-density shadow posterior to the spleen.

**Figure 2.**
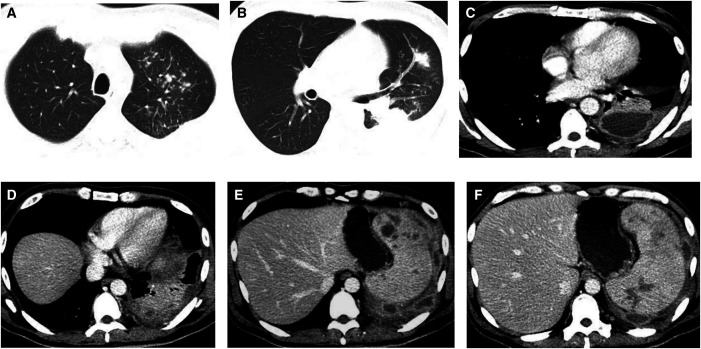
The thoracic and abdominal computed tomography of 2 months before admission. (**A–D**) Exacerbated lung lesions, which predominantly manifested as nodular changes in the left upper lobe, along with persistent left pleural effusion. (**E**, **F**) The spleen was enlarged and contained multiple irregular, low-density areas with heterogeneous enhancement.

A 40-year-old male employee residing in Sanya City, Hainan Province, presented to our hospital with recurrent chest pain, cough, and abdominal discomfort that had persisted for 11 months and exacerbated over the 2 months prior to presentation. His medical history included poorly controlled type 2 diabetes mellitus, diagnosed 1 year prior, with no hypoglycemic therapy administered. Eleven months before admission, he began to experience left lower chest pain that was aggravated by deep inspiration and coughing. He also developed a cough with expectoration and pain in the left upper abdomen. Computed tomography (CT) of the thoracic and abdominal regions revealed dispersed infectious lesions within the left lung, predominantly located in the lower lobe, accompanied by partial consolidation. In addition, there was an encapsulated pleural effusion on the left side, multiple abnormal, weakly enhanced shadows in the spleen, and a low-density shadow posterior to the spleen (Figure 1). Owing to the mild and tolerable nature of the patient’s symptoms, coupled with a demanding work schedule, the patient did not undergo further evaluation or treatment at that time. Two months before admission, the patient presented with worsening chest and abdominal pain, increased cough frequency, expectoration of small amounts of yellow mucoid sputum, and weight loss. Upon reevaluation, thoracic and abdominal CT scans demonstrated worsening of the pulmonary lesions, primarily presenting as nodular alterations in the left upper lobe. In addition, persistent left pleural effusion and splenic abnormalities, including splenomegaly, were observed (Figure 2). His symptoms persisted despite treatment with levofloxacin for half a month as anti-infective medication.

During the physical examination, a reduction in tactile vocal fremitus was observed in the left lower lung region. Percussion of the same area revealed dullness, accompanied by decreased breath sounds. Scattered moist rales were audible in the left lung of the patient. The heart rate was 129 beats per minute with a regular rhythm. Tenderness with mild rebound was observed in the left upper and mid-abdominal regions. The spleen was palpable five fingerbreadths below the costal margin at the left midclavicular line.

The results of the main laboratory tests are presented in Table 1. Pleural effusion was characterized as purulent, with glucose levels in the pleural effusion measuring <3.3 mmol/L and lactate dehydrogenase (LDH) exceeding 1,000 U/L. The leukocytes observed in the pleural effusion were predominantly neutrophils. Aseptically collected pleural fluid was centrifuged; the sediment was inoculated onto Columbia blood agar and incubated at 37°C. Suspected colonies were identified using the VITEK 2 Compact GN card (bioMérieux; Marcy-l’Étoile, France). The system confirmed *B. pseudomallei* with 99% probability. Consequently, the pleural effusion was diagnosed as empyema. Antimicrobial susceptibility testing was performed using the VITEK 2 Compact automated system (bioMérieux) with AST N335 cards. Minimum inhibitory concentrations (MICs) were interpreted according to the CLSI M45 break points for *B. pseudomallei* (where available). The tested antibiotics included doxycycline, imipenem, ceftazidime, and trimethoprim/sulfamethoxazole. Detailed antimicrobial sensitivity results are presented in Table 2.

**Table 1:** Laboratory parameters

Laboratory Parameters	Results	Reference Values
White blood cell count, cells/mm^3^	12,690	3,500–9,500
Neutrophils, %	85	40–75
Hemoglobin, g/dL	9.2	12.0–16.0
Procalcitonin, ng/mL	0.082	<0.46
C-reactive protein, mg/L	89.67	≦6.00
Blood glucose, mmol/L	14.05	3.9–6.1
Glycated hemoglobin, %	8.8	4.0–6.0
Albumin, g/L	31.9	40–55
Appearance of the pleural effusion	Purulent	–
Total protein of the pleural effusion, g/L	71.2	–
White blood cell count of the pleural effusion, cells/mm^3^	145,940	–
Neutrophils of the pleural effusion, %	78	–
Glucose of the pleural effusion, mmol/L	1.81	–
Lactate dehydrogenase of the pleural effusion, U/L	35,240	–

**Table 2:** Antimicrobial susceptibility results

Drug Name	Minimum Inhibitory Concentration (*µ*g/mL)	Sensitivity
Doxycycline	≦1	Sensitive
Imipenem	≦1	Sensitive
Ceftazidime	≦1	Sensitive
Trimethoprim/sulfamethoxazole	≦2/38	Sensitive

**Figure 3.**
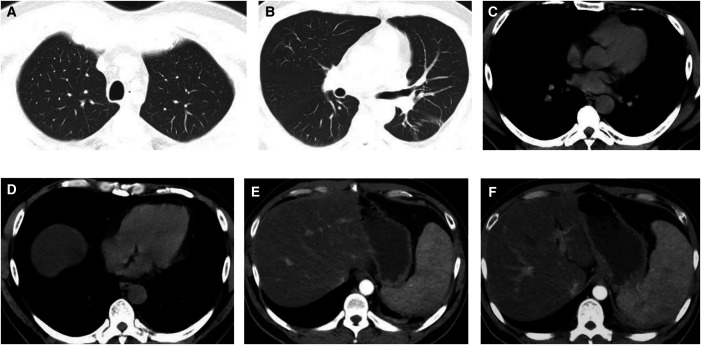
Six-month follow-up computed tomography of the chest and abdomen. (**A–D**) The scattered infectious lesions in the left lung had largely resolved, and the left pleural effusion had essentially disappeared. (**E**, **F**) Multiple abnormal low-density shadows in and around the spleen were essentially resolved.

The patient received intensive intravenous ceftazidime for 2 weeks, followed by an eradication phase of oral trimethoprim/sulfamethoxazole for 3 months. A 6-month follow-up CT scan revealed that the scattered infectious lesions in the left lung had largely resolved, and the left pleural effusion had essentially disappeared. Multiple abnormal low-density shadows in and around the spleen had also resolved (Figure 3).

## DISCUSSION

*B. pseudomallei* is a Gram-negative aerobic bacillus that resides saprophytically in soil and water, particularly in paddy fields. The principal routes of transmission are direct contact through damaged skin, inhalation via the respiratory tract, and ingestion through the digestive tract.^7^ The pathogenicity of *B. pseudomallei* is driven primarily by virulence factors and exotoxins, which allow the bacterium to adhere to, invade, and multiply within host cells. Intracellular survival serves as a key mechanism of immune evasion, which likely underlies latent and chronic infections observed in melioidosis.^8^ *B. pseudomallei* is most abundant in soil at depths of ≥10 cm from the surface.^9^ A prediction model showed that high rainfall, soil types (e.g., anthrosol and acrisol), and salinity were all strongly correlated with the presence of *B. pseudomallei* in the environment.^10^ Such conditions are hypothesized to bring *B. pseudomallei* to the surface as the water level rises. It proliferates on the surface; therefore, farmers, fishermen, and men are susceptible populations. Diabetes mellitus is the most common risk factor predisposing individuals to melioidosis, which is found in >50% of all patients with melioidosis worldwide.^11^ Hazardous alcohol consumption, chronic kidney disease, chronic lung disease, immunosuppressive therapy (most commonly corticosteroids), and thalassemia with iron overload are also well-recognized risk factors for melioidosis.

Melioidosis can affect multiple organ systems and typically manifests as localized abscesses, bacteremia, and sepsis. *B. pseudomallei* infections can present as acute or chronic inflammation. The latter is often characterized by granuloma formation. Chronic infection is associated with genetic variation in pathogens, adaptation to the environment, and immune escape.^8^ The clinical features of chronic infection are chronic abscesses, which are very similar to those associated with tuberculosis, fungal infections, or tumors.^12^ Host responses to melioidosis and tuberculosis are similar, both dominated by interferon-signaling pathways.^13^ Acute melioidosis pneumonia frequently manifests on imaging as multiple small nodules, alveolar infiltration, and consolidation, which may rapidly progress to cavitation. These nodules and consolidations generally appear first in the upper lobes and may quickly enlarge, coalesce, and form cavities.^14^ In cases of subacute or chronic pulmonary melioidosis, the majority of patients exhibit an indolent disease course. Subacute and chronic pulmonary melioidosis may present with mixed nodules or patchy opacities with slower progression in the subacute form.^15^ On the other hand, chronic pulmonary melioidosis can manifest as cavitary lesions.^16^

Empyema is commonly associated with pathogens, such as *Staphylococcus aureus*, *Streptococcus*, Enterobacterales, *Pseudomonas* spp., anaerobes, mycobacteria, and fungi.^17^ However, empyema caused by *B. pseudomallei* is infrequently reported. Our study showed that pleural effusion due to *B. pseudomallei* infection is typically purulent and characterized by neutrophil-dominant inflammation. However, it can also present with lymphocyte-predominant inflammation. *B. pseudomallei* possesses capsular exopolysaccharides. These capsular exopolysaccharides induce the production of cytokines, such as tumor necrosis factor, interleukin (IL)-8, and IL-10, from leukocytes, which are involved in the chemotaxis of lymphocytes into the pleural cavity.^18^ Therefore, melioidosis should be considered in the differential diagnosis of lymphocytic pleural effusions, in addition to tuberculosis or malignancy, especially in endemic areas where tuberculosis is commonly observed.

The lung is the most frequently impacted organ by melioidosis.^19^^,^^20^ The spleen is the most commonly affected extrapulmonary visceral organ.^15^^,^^21^ Splenic lesions occurring along with an infective focus in the body would favor the diagnosis of melioidosis.^20^ Case reports indicate that splenic melioidosis can present as a latent asymptomatic infection^22^ or as a chronic condition,^23^^,^^24^ highlighting that splenic abscesses can be a manifestation of chronic melioidosis. Splenic abscesses attributable to *B. pseudomallei* typically manifest as multiple, small, and discrete single or multiple multiloculated lesions, as well as subcapsular collections, with or without perisplenic extension.^15^ The findings reported in the literature align with the imaging results of splenic abscesses as delineated in this article. Surgical drainage of large abscesses is indicated but is usually not required for multiple small liver and splenic abscesses.^8^ Surgical intervention is technically challenging and high-risk, and small abscesses may be missed, which increases the risk of recurrence. Antibiotic therapy alone would be sufficient in the treatment of melioidosis with splenic abscesses.^25^

*B. pseudomallei* is difficult to treat because it is intrinsically resistant to multiple classes of antimicrobials. Few effective antibiotics are available for treatment, including ceftazidime, imipenem, meropenem, amoxicillin/clavulanate, doxycycline, and trimethoprim/sulfamethoxazole.^3^

## CONCLUSION

Melioidosis is a neglected tropical disease that remains underdiagnosed in many geographical areas. Delayed diagnosis contributes to increased mortality and morbidity. Therefore, health care professionals should maintain a high level of vigilance for atypical etiologies of pneumonia or parapneumonic effusion in individuals with compromised immune systems, particularly those with diabetes, as well as in individuals residing in or traveling to regions where the disease is endemic. High vigilance is especially required when standard intravenous antibiotic treatments, administered according to established guidelines for community-acquired pneumonia, fail to produce satisfactory outcomes. Multiple organ infections, notably with splenic abscesses, are critical diagnostic indicators of melioidosis.
